# Sequencing of the Complete Mitochondrial Genome of the Big Brown Mactra Clam, *Mactra grandis* (Venerida: Mactridae)

**DOI:** 10.3390/ani14091376

**Published:** 2024-05-03

**Authors:** Peizhen Ma, Zhihong Liu, Zhuanzhuan Li, Xiujun Sun, Liqing Zhou, Xiangyu Wu, Biao Wu

**Affiliations:** 1 State Key Laboratory of Mariculture Biobreeding and Sustainable Goods, Yellow Sea Fisheries Research Institute, Chinese Academy of Fishery Sciences, Qingdao 266071, China; mapz@ysfri.ac.cn (P.M.); liuzh@ysfri.ac.cn (Z.L.); lizz@ysfri.ac.cn (Z.L.); xjsun@ysfri.ac.cn (X.S.); zhoulq@ysfri.ac.cn (L.Z.); 2 Key Laboratory of Sustainable Development of Marine Fisheries, Ministry of Agriculture and Rural Affairs, Yellow Sea Fisheries Research Institute, Chinese Academy of Fishery Sciences, Qingdao 266071, China; 3 Hainan Provincial Key Laboratory of Tropical Maricultural Technology, Hainan Academy of Ocean and Fisheries Sciences, Haikou 571126, China

**Keywords:** genetic distance, Mactridae, mitochondrial genome, phylogeny, selective pressure

## Abstract

**Simple Summary:**

Mitochondrial genomes have become a powerful tool for studying molecular genetics and phylogeny of mollusks. In this study, the complete mitochondrial genome of *Mactra grandis* was characterized for the first time. The newly sequenced mitochondrial genome fits the typical composition pattern of mollusks with 37 functional genes. Among the Mactridae species with reported mitochondrial genomes, *Mactra grandis* has the closest relationship with *Mactra cygnus*. The gene arrangement, genetic distance, and selective pressure of protein-coding genes among *Mactra* species were also analyzed. This study provides a molecular basis for taxonomy and germplasm research on Mactridae species.

**Abstract:**

Mitochondrial genomes are playing an increasingly important role in molluscan taxonomy, germplasm, and evolution studies. The first complete mitochondrial genome of the commercial big brown mactra clam, *Mactra grandis*, was characterized using Illumina next-generation sequencing in this study. The 17,289 bp circular genome has a typical gene organization of 13 protein-coding genes (PCGs), 2 rRNAs, and 22 tRNAs, with an obvious (A + T)-bias of 64.54%. All PCGs exhibited a homogeneous bias in nucleotide composition with a (A + T)-bias, a positive GC skew, and a negative AT skew. Results of phylogenetic analysis showed that *Mactra grandis* was most closely related to *Mactra cygnus*. The functional gene arrangement of the two species was identical but different from other *Mactra* species. The congeneric relationships among *Mactra* species were demonstrated by genetic distance analysis. Additionally, the selective pressure analysis suggested that *cox1* was highly efficient for discriminating closely related species in genus *Mactra*, while *nad2* was the most appropriate marker for population genetic analysis.

## 1. Introduction

With the continuous decrease in the cost of sequencing high-quality genomes, an increasing number of mitochondrial genomes for mollusks have been reported. These contributions have significantly enriched studies in taxonomy, germplasm research, and investigations into adaptive evolution [1,2,3,4]. On one hand, molluscan mitogenomes offer valuable molecular insights for biological taxonomy research, encompassing conserved sequences of functional genes and gene arrangements among closely related species [5,6,7]. On the other hand, mitochondrial genomes have a faster evolutionary rate than nuclear genomes and contain appropriate gene markers, mitochondrial single nucleotide polymorphisms, and mutations, which can be used for population genetic diversity and germplasm evaluation [8,9,10]. Nonetheless, there are still far too few species with sequenced mitogenomes considering the tens of thousands of existing mollusks in the world [11]. 

The family Mactridae, classified by Lamarck in 1809, encompasses approximately 150 species distributed globally. Commonly characterized by their thin and fragile shells, these bivalves are referred to as surf or trough clams [12,13]. Synonyms and taxa rearrangements are quite common in Mactridae species because their shell coloration and forms tend to change with the environment [14]. Mitochondrial genomes have been proven effective in recognizing taxonomic and phylogenetic problems in Mactridae species. However, previous phylogenetic data were insufficient to address these issues adequately [15]. *Mactra grandis* (Gmelin, 1791), synonymous with *Mactra mera* (Reeve, 1854), is an edible large mactrid species distributed in tropical Indo-Pacific regions [14]. Known as the big brown mactra clam, *Mactra grandis* has shells ranging from 6 to 7 cm and is considered the most commonly encountered member of the family Mactridae in Singapore [16]. While the type specimen of *Mactra mera* was collected in China as early as 1854 [17], modern records for this species only began in 1960 [18]. Despite its historical and contemporary significance, molecular data for *Mactra grandis* remain virtually absent.

The mitochondrial genome of *Mactra grandis* was characterized for the first time in this study. The aims were to (1) provide the first complete mitogenome of the commercial species and (2) verify the classification among *Mactra* species.

## 2. Materials and Methods

### 2.1. Sample Collection and DNA Extraction

A specimen of *Mactra grandis* (shell length 6.67 cm, height 4.78 cm, and width 3.06 cm) was collected on 9 August 2023 from the Li’an Bay, Hainan Province (18°25.387′ N, 110°1.039′ E). The adductor muscle was exclusively selected for DNA extraction to mitigate potential confounding factors associated with the doubly uniparental inheritance observed in mussels and clams [19]. The rest of the specimen was preserved in 95% ethanol and deposited in the State Key Laboratory of Mariculture Biobreeding and Sustainable Goods, Yellow Sea Fisheries Research Institute, Chinese Academy of Fishery Sciences. Total genomic DNA was extracted using the TIANamp Marine Animals DNA Kit (DP324-03; Tiangen Biotech (Beijing), Beijing, China), following the manufacturer’s protocol.

### 2.2. Sequencing, Assembly, and Genome Annotation

The genomic library was constructed with the whole-genome shotgun strategy and sequenced on the Illumina NovaSeq platform (Illumina, San Diego, CA, USA) at Shanghai Personal Biotechnology Co., Ltd. (Shanghai, China) by using the 2 × 150 bp paired-end sequencing mode and with an insert size of 400 bp. The software NGS QC Toolkit v2.3.3 [20] was used for quality control of raw data. Then, GetOrganelle v1.7.7.0 [21] and SPAdes v3.9.0 [22] were employed for the de novo assembly to construct contig and scaffold sequences. BLASTN was conducted in the NCBI nucleotide database using Blast v2.2.31+. And finally, Mummer v3.1 [23] and Pilon v1.18 [24] were used to fill gaps between contigs.

The complete mitogenome sequence was uploaded to the MITOS2 Web Server (http://mitos2.bioinf.uni-leipzig.de/index.py, accessed on 15 October 2023) for functional annotation [25]. The genetic code was selected as “5 Invertebrate Mitochondrial”. The boundaries of PCGs were determined by an ORF finder (https://www.ncbi.nlm.nih.gov/orffinder, accessed on 15 October 2023) and corrected manually by comparison with genes from the same family [15]. The mitochondrial genome circular map was drawn using the Proksee [26]. 

### 2.3. Genome Composition and Codon Usage

MEGA 7.0 software [27] was applied to calculate the nucleotide base composition of the newly sequenced mitogenome. GC skew was determined using the following formulae: GC skew= (G − C)/ (G + C) and AT skew = (A − T)/ (A + T), where G, C, A, and T represent the frequency of each nucleotide base. PhyloSuite v1.1.16 [28] was used to analyze the relative synonymous codon usage (RSCU) of the mitogenome.

### 2.4. Phylogenetic Analysis and Gene Arrangement

Phylogenetic relationships within the family Mactridae were determined based on the datasets of 13 PCGs and 2 rRNAs by PhyloSuite [28], with *Donax trunculus* from the family Donacidae and *Mercenaria mercenaria* from the family Veneridae being the outgroup. MAFFT was employed to align the PCGs under codon mode and rRNAs under normal mode independently [29]. Then, all PCG and rRNA alignments files were concatenated into a data matrix. The best partitioning scheme and evolutionary models for 15 pre-defined partitions were selected using PartitionFinder2 [30], with greedy algorithm and AICc criterion.

Phylogenetic trees were reconstructed using Bayesian inference (BI) and maximum likelihood (ML) analyses. Bayesian inference phylogenies were inferred using MrBayes 3.2.6 [31] under partition model (2 parallel runs, 200,000 generations), in which the initial 25% of sampled data were discarded as burn-in. Maximum likelihood phylogenies were inferred using IQ-TREE [32] under an edge-linked partition model for 5000 ultrafast bootstraps [33], as well as the Shimodaira–Hasegawa–like approximate likelihood-ratio test [34]. Phylogenetic trees and gene arrangements were visualized using the Interactive Tree of Life [35]. The branch support values of Bayesian posterior probabilities (PP) and the maximum likelihood bootstrap support values (BS) were shown on the trees. The CREx algorithm was employed to reconstruct the putative gene order rearrangement events that might have transpired within the genus *Mactra* [36].

### 2.5. Selective Pressure and Genetic Distance Analysis

Based on the phylogenetic results, the selective pressure on PCGs in the two main *Mactra* clades was analyzed, respectively. Software PhyloSuite [28] was employed to perform the preparation of the mitochondrial PCG sequences. The sequences were aligned in batches with MAFFT [29] using ‘-auto’ strategy and codon alignment mode. The alignments were refined using the codon-aware program MACSE v. 2.03 [37], which preserved the reading frame and allowed incorporation of sequencing errors or sequences with frameshifts. Ambiguously aligned fragments of 13 alignments were removed in batches using Gblocks [38]. DnaSP6 software [39] was then used to calculate the nonsynonymous substitution rate (Ka) and synonymous substitution rate (Ks) of each PCG. Given their widespread utilization as genetic markers in population, phylogeny, and evolution studies of bivalves, *cox1* and *16S* were chosen alongside PCG nad2, which experienced the least selective pressure in this investigation, to assess the genetic distances between the two primary clades within the genus *Mactra* [8,40,41]. The genetic distances were analyzed using the Kimura 2-parameter model in MEGA 7.0 [29] to elucidate their taxonomic relationships, with only one sequence utilized per species.

## 3. Results and Discussion

### 3.1. General Features of Mitogenome

**Figure 1:**
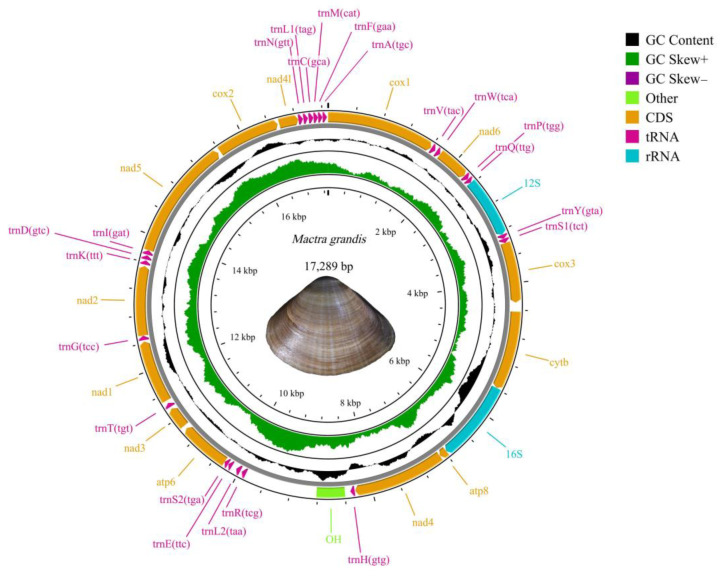
The mitochondrial genome map of *Mactra grandis,* collected in Li’an Bay, Hainan, China.

The raw sequencing data for *Mactra grandis* mitogenome included 19,544,316 reads and a total base of 2,951,191,716 bp, with Q20 and Q30 values being 96.72% and 94.21%, respectively. Altogether, 19,344,846 high-quality reads with 2,913,422,642 bp were obtained, accounting for 98.97% of the whole reads. After assembly and annotation, the complete mitochondrial genome of *Mactra grandis* showed a double-stranded circular molecule structure and had 17,289 bp in length (GenBank accession no. OR897711, Figure 1). The mitogenome had the typical gene organization of Mactridae [15], including 13 PCGs (*cox1*, *cox2*, *cox3*, *cytb*, *nad1*, *nad2*, *nad3*, *nad4*, *nad4l*, *nad5*, *nad6*, *atp6*, and *atp8*), two rRNA genes (*12S* and *16S*), and 22 tRNAs (Table 1). The nucleotide composition was 24.90% for A, 23.32% for G, 12.14% for C, and 39.64% for T, exhibiting an obvious (A + T)-bias of 64.54%. All the functional genes were encoded on the heavy strand.

### 3.2. Protein-Coding Genes

Variation and heterogeneity of DNA base compositions among species or gene fragments were the results of evolutionary adaptation to the environment [42]. Interestingly, all 13 PCGs of *Mactra grandis* mitogenome exhibited a homogeneous bias in nucleotide composition with a (A + T)-bias from 61.45% (*nad5*) to 72.81% (*atp8*), a positive GC skew from 0.2070 (*cytb*) to 0.5484 (*atp8*), and a negative AT skew from −0.4171 (*nad4l*) to −0.2028 (*cox2*). *Cox1*, *nad3*, and *nad4* had GTG and *nad1* had ATA at the sequence start, while the other 9 PCGs had ATG at the sequence start. All the PCGs except *nad4l*, which was truncated with nucleotide T, had TAA or TAG at the sequence end.

**Figure 2:**
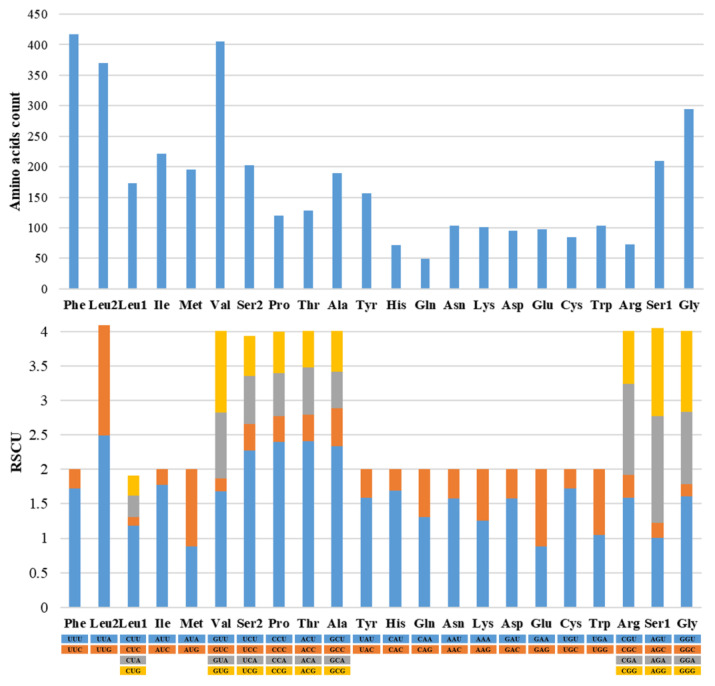
Amino acids count and relative synonymous codon usage (RSCU) of *Mactra grandis* mitochondrial genome.

The relative synonymous codon usage (RSCU) of *Mactra grandis* mitogenome indicated Phe, Val, and Leu were the three most frequently used amino acids (417, 405, and 370 counts, respectively) (Figure 2). Consistent with other mactrid mitogenomes, NNU and NNA were dominant in most codons, indicating a preferred sequence ending with A or T. UUA-Leu2, UCU-Ser2, CCU-Pro, ACU-Thr, and GCU-Ala were the five most frequently used codons. All five codons had RSCU values over 2.

### 3.3. Ribosomal RNAs and Transfer RNAs

The lengths of *12S* and *16S* ribosomal RNAs of *Mactra grandis* were 895 bp and 1193 bp, respectively. The *12S* rRNA was located between *trnP* and *trnY*, while the *16S* rRNA was located between *cytb* and *atp8*. The AT base contents for *12S* and *16S* were 64.43% and 67.79%, respectively, indicating AT biases. Both rRNAs showed positive GC skew (0.2366 and 0.2727 for *12S* and *16S*, respectively). However, the *12S* showed a negative AT skew (−0.0138) while the *16S* showed a positive AT skew (0.0050).

The 22 mitochondrial tRNAs of *Mactra grandis* ranged from 61 bp (*trnH* and *trnS2*) to 70 bp (*trnC*). All the tRNAs showed positive GC skews, ranging from 0.0526 in *trnE* to 0.5000 in *trnD*. Except for *trnL1*, for which the AT skew was positive (0.0667), all tRNAs showed negative AT skews, ranging from −0.2973 in *trnS2* to −0.0233 in *trnW*. All tRNAs showed a typical cloverleaf model. The origin for the heavy strand replication (*OH*), 416 bp, was located between *trnH* and *trnR*.

### 3.4. Phylogeny and Gene Arrangement Analysis

**Figure 3:**
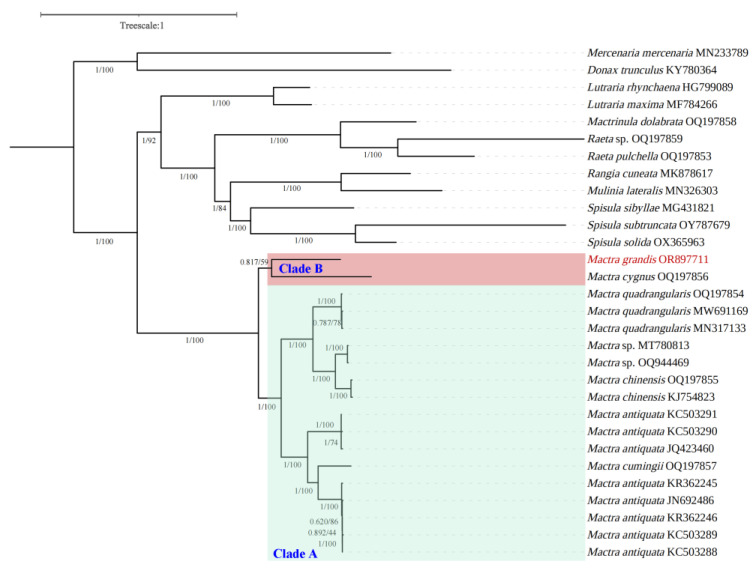
Phylogenetic tree of Mactridae species based on 13 protein-coding genes and 2 rRNAs, with *Mercenaria mercenaria* and *Donax trunculus* being the outgroup. Numbers near the nodes are branch support values of Bayesian posterior probabilities, followed by maximum likelihood bootstrap support values. Mitogenome sequence obtained in this study was marked in red.

The best partitioning scheme in this study had 164 params, 18,324 sites, and 10 subsets, with InL and AICc being −221,561.8095703125 and 443,454.599481, respectively. The best evolutionary models were listed in Table 2. Phylograms derived from ML and BI analyses had identical topologies, suggesting that the family Mactridae was subdivided into two main clades (Figure 3, PP = 1, BS = 100). One clade covered only *Mactra* species, including eighteen mitochondrial genome sequences from 7 species and a cryptic species in *Mactra antiquata*. The newly sequenced species, *Mactra grandis*, clustered with *Mactra cygnus*, demonstrating a sister relationship (Clade B). Other *Mactra* species clustered to Clade A, including *Mactra quadrangularis*, *Mactra* sp., *Mactra chinensis*, *Mactra antiquata*, the cryptic species in *Mactra antiquata*, and *Mactra cumingii*, with the first five species having two or more sequences. Although markedly morphologically different from *Mactra antiquata*, *Mactra cumingii* occupied the phylogenetic position to divide the *Mactra antiquata* mitogenomes into two types, which supported the existence of a cryptic species in *Mactra antiquata* [2,43]. The other ten Mactridae species from 6 genera constituted another main clade, including four genera from subfamily Mactrinae (*Mactrinula*, *Rangia*, *Mulinia*, and *Spisula*), one genus from subfamily Lutrariinae (*Lutraria*), and one family from subfamily Anatinellidae (*Raeta*). Our results provided support for removing *Mactrinula*, *Rangia*, *Mulinia*, and *Spisula* out from subfamily Mactrinae [15]. Two *Raeta* species first clustered with *Mactrinula dolabrata*, prompting the attribution of *Raeta* to family Mactridae instead of Anatinellidae [44]. Results also showed that all congeneric species in Mactridae clustered together, indicating closer relationships.

**Figure 4:**
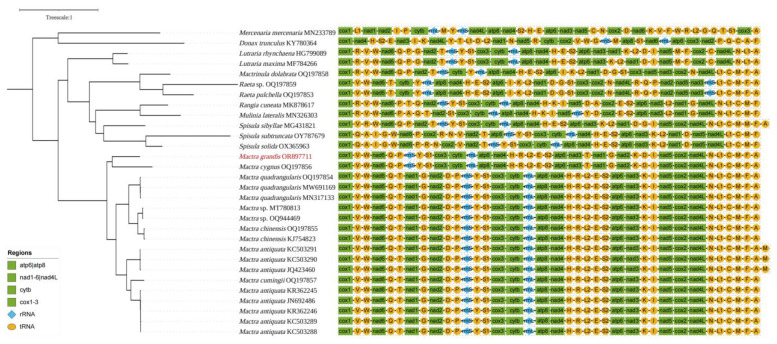
Gene arrangements of mitogenomes in Mactridae. Mitogenome sequence obtained in this study was marked in red.

Mitochondrial gene arrangements among closely related mollusks were usually highly conserved, although gene rearrangements could be relatively frequent in Mollusca [45,46,47]. In this study, most Mactridae species had 22 tRNAs, besides *Spisula sibyllae* (MG431821) and *Mactra antiquata* (KC503290, KC503291, and JQ423460), which had a duplication of *trnM* (Figure 4). Although the genus *Mactra* was shown as a monophyletic group, two types of gene arrangement were observed within the genus. Clade A and Clade B had independent gene orders. The differences involved the translocations of two long gene chains: -*trnT*-*nad1*-*trnG*-*nad2*-*trnD*- and –*trnP*-*12S*-*trnY*-*trnS1*-*cox3*-*cytb*-*16S*-*atp8*- *nad4*-*trnH*-*trnR*-*trnL2*-*trnE*-*trnS2*-*atp6*-*nad3*-*trnK*. CREx analysis indicated the occurrence of a tandem duplication random loss (TDRL) event from *Mactra grandis* to *Mactra quadrangularis*. This difference put forward the question of whether the two clades belonged to one genus. Additionally, three species in genus *Spisula* also showed differences in not only generic makeup but also gene orders. However, in genera *Lutraria* and *Raeta*, gene arrangements were highly conserved.

### 3.5. Genetic Comparison within Genus Mactra

To further clarify the relationships between Clade A and Clade B, selective pressure and genetic distance analyses were conducted in this study. The results of selective pressure analysis within the two clades in *Mactra* showed that all PCGs had Ka/Ks < 1 (Table 3), indicating purifying selection in *Mactra* species. The minimum Ka/Ks value of *Cox1* was 0.02989 in Clade A and 0.07025 in clade B, indicating that mitochondrial *cox1* would be highly efficient for discriminating closely related species in genus *Mactra* [48,49,50]. Except for the *atp8* in Clade A, *nad2* showed the highest Ka/Ks values in both clades, suggesting that *nad2* was subject to the least selective pressure among the 12 PCGs and had the highest variation. This makes *nad2* the most suitable marker for population genetic analysis [8] and may lead to gene chain translocation between the two clades. The genetic distances within Clade A were determined to be 0.17266, 0.29388, and 0.13860 based on the *cox1*, *nad2*, and *16S* rRNA, respectively. However, the genetic distances between the two clades were comparable with that within Clade B based on *cox1* (0.21858 < 0.22756), *nad2* (0.49023 < 0.49587), and *16S* rRNA (0.26458 < 0.28222). The results were consistent with the interspecific genetic distances within *Mactra*, based on either *cox1* (0.085 ~ 0.284) or *16S* (0.014 ~ 0.271) [48]. As a result, species from both Clade A and Clade B should belong to the same genus.

## 4. Conclusions

The complete mitochondrial genome of *Mactra grandis* is a circular molecule of 17,289 bp, with 13 protein-coding genes, 2 rRNAs, and 22 tRNAs. All protein-coding genes in *Mactra grandis* mitogenome exhibited a homogeneous bias in nucleotide composition with a (A + T)-bias, a positive GC skew, and a negative AT skew. Among the Mactridae species with mitogenomes reported, *Mactra grandis* is the most closely related to *Mactra cygnus* in terms of both the mitochondrial molecular dendrogram and the functional gene arrangement. By contrast, other *Mactra* species share another gene arrangement, with differences in the translocations of two long gene chains: -*trnT*-*nad1*-*trnG*-*nad2*-*trnD*- and –*trnP*-*12S*-*trnY*-*trnS1*-*cox3*-*cytb*-*16S*-*atp8*-*nad4*-*trnH*-*trnR*- *trnL2*-*trnE*-*trnS2*-*atp6*-*nad3*-*trnK*. Still, the congeneric relationships among *Mactra* species are determined. The selective pressure analysis of mitochondrial protein-coding genes further suggests that *cox1* is highly efficient for discriminating closely related species in genus *Mactra* and that *nad2* is the most appropriate marker for population genetic analysis.

## Figures and Tables

**Table 1:** Organization of the mitochondrial genome of *Mactra grandis*.

Feature	Position		Length (bp)	Codon		Anticodon	Intergenic Region	GC Skew	AT Skew	Strand
	From	To		Start	Stop					
*cox1*	1	1596	1596	GTG	TAA		8	0.2096	−0.3097	+
*trnV*	1605	1671	67			TAC	21	0.1304	−0.0455	+
*trnW*	1693	1758	66			TCA	3	0.3043	−0.0233	+
*nad6*	1762	2238	477	ATG	TAA		2	0.2593	−0.3778	+
*trnQ*	2241	2308	68			TTG	2	0.3548	−0.2432	+
*trnP*	2311	2373	63			TGG	0	0.4737	−0.0455	+
*12S*	2374	3268	895				0	0.2366	−0.0138	+
*trnY*	3269	3332	64			GTA	0	0.2308	−0.1579	+
*trnS1*	3333	3397	65			TCT	0	0.3600	−0.2500	+
*cox3*	3398	4288	891	ATG	TAA		133	0.3046	−0.3145	+
*cytb*	4422	5582	1161	ATG	TAA		0	0.2070	−0.2474	+
*16S*	5583	6775	1193				0	0.2727	0.0050	+
*atp8*	6776	6889	114	ATG	TAA		3	0.5484	−0.2771	+
*nad4*	6893	8248	1356	GTG	TAA		8	0.2660	−0.2974	+
*trnH*	8257	8320	64			GTG	80	0.3846	−0.0417	+
*OH*	8401	8816	416				1079	0.3797	−0.1528	+
*trnR*	9896	9962	67			TCG	25	0.2857	−0.1282	+
*trnL2*	9988	10,054	67			TAA	73	0.4194	−0.1111	+
*trnE*	10,128	10,187	60			TTC	4	0.0526	−0.1600	+
*trnS2*	10,192	10,252	61			TGA	0	0.4167	−0.2973	+
*atp6*	10,253	10,999	747	ATG	TAG		50	0.2615	−0.2977	+
*nad3*	11,050	11,361	312	GTG	TAG		30	0.2593	−0.3529	+
*trnT*	11,392	11,458	67			TGT	41	0.3333	0.0000	+
*nad1*	11,500	12,429	930	ATA	TAA		20	0.3275	−0.3573	+
*trnG*	12,450	12,516	67			TCC	1	0.3333	−0.0698	+
*nad2*	12,518	13,543	1026	ATG	TAA		20	0.3277	−0.4137	+
*trnK*	13,564	13,627	64			TTT	13	0.2800	−0.2308	+
*trnD*	13,641	13,704	64			GTC	9	0.5000	−0.2500	+
*trnI*	13,714	13,780	67			GAT	1	0.2727	−0.1176	+
*nad5*	13,782	15,566	1785	ATG	TAG		35	0.3422	−0.3406	+
*cox2*	15,602	16,540	939	ATG	TAG		22	0.4586	−0.2028	+
*nad4l*	16,563	16,851	289	ATG	T--		0	0.4667	−0.4171	+
*trnN*	16,852	16,919	68			GTT	6	0.3333	−0.0638	+
*trnL1*	16,926	16,992	67			TAG	10	0.4545	0.0667	+
*trnC*	17,003	17,072	70			GCA	3	0.2500	−0.0435	+
*trnM*	17,076	17,141	66			CAT	1	0.1852	−0.0256	+
*trnF*	17,143	17,206	64			GAA	2	0.4783	−0.0244	+
*trnA*	17,209	17,272	64			TGC	17	0.2727	−0.1429	+

**Table 2:** Partitions and evolutionary models selected by PartitionFinder2 for phylogenetic analyses.

Subset	Best Model	Sites	Partition Names
1	GTR+I+G	1815	atp8_mafft, atp6_mafft, nad6_mafft
2	GTR+G	2205	cox1_mafft
3	GTR+I+G	1941	cox2_mafft
4	GTR+I+G	3660	nad3_mafft, nad4_mafft, cox3_mafft, nad4l_mafft
5	GTR+I+G	1479	cytb_mafft
6	GTR+G	1071	nad1_mafft
7	GTR+I+G	1206	nad2_mafft
8	GTR+I+G	1944	nad5_mafft
9	GTR+I+G	1293	12S_mafft
10	GTR+I+G	1710	16S_mafft

**Table 3:** The evolutionary constraint (Ka/Ks) analyses of 13 mitochondrial protein-coding genes in two clades of genus *Mactra*. Ka: nonsynonymous substitution rate; Ks: synonymous substitution rate calculations.

Genes	Clade A				Clade B			
	bp	Ka	Ks	Ka/Ks	bp	Ka	Ks	Ka/Ks
*atp6*	741	0.04997	0.57542	0.08684	741	0.13856	0.68861	0.20122
*atp8*	111	0.07500	0.30536	0.24561	108	0.08219	0.56934	0.14436
*cox1*	1569	0.01515	0.50683	0.02989	1572	0.04666	0.66421	0.07025
*cox2*	975	0.11010	0.55347	0.19893	906	0.21560	0.78303	0.27534
*cox3*	888	0.04510	0.51489	0.08759	888	0.11612	0.66011	0.17591
*cytb*	1278	0.05887	0.53214	0.11063	1149	0.09115	0.68110	0.13383
*nad1*	888	0.03690	0.48333	0.07635	882	0.13136	0.65703	0.19993
*nad2*	1017	0.11320	0.50772	0.22296	1017	0.24745	0.68908	0.35910
*nad3*	354	0.08061	0.61969	0.13008	300	0.15839	0.73479	0.21556
*nad4*	1211	0.06225	0.58931	0.10563	1188	0.17878	0.70473	0.25369
*nad4l*	288	0.07549	0.48394	0.15599	288	0.15498	0.73284	0.21148
*nad5*	1782	0.11982	0.57578	0.20810	1749	0.23136	0.68076	0.33986
*nad6*	471	0.09472	0.52443	0.18062	456	0.19788	0.70279	0.28156

## Data Availability

The mitochondrial genome of *Mactra grandis* is available from GenBank under the accession no. OR897711.
